# The expression pattern, subcellular localization and function of three sterol 14α-demethylases in *Aspergillus oryzae*

**DOI:** 10.3389/fgene.2023.1009746

**Published:** 2023-01-23

**Authors:** Qi Jin, Ganghua Li, Kunhai Qin, Yitong Shang, Huanhuan Yan, Hongliang Liu, Bin Zeng, Zhihong Hu

**Affiliations:** ^1^ Jiangxi Key Laboratory of Bioprocess Engineering, College of Life Sciences, Jiangxi Science and Technology Normal University, Nanchang, China; ^2^ Hubei Key Laboratory of Edible Wild Plants Conservation and Utilization, Hubei Normal University, Huangshi, China; ^3^ College of Pharmacy, Shenzhen Technology University, Shenzhen, China

**Keywords:** Aspergillus oryzae, sterol 14α-demethylase, ergosterol, subcellular localization, function

## Abstract

Sterol 14α-demethylase catalyzes lanosterol hydroxylation, which is one of the key reactions in the biosynthetic pathway of sterols. There is only one sterol 14α-demethylases gene named *Erg11* in *Saccharomyces cerevisiae* genome. In this study, three sterol 14α-demethylases genes named *AoErg11A, AoErg11B* and *AoErg11C* were identified in *Aspergillus oryzae* genome through bioinformatics analysis. The function of these three genes were studied by yeast complementation, and the expression pattern/subcellular localization of these genes/proteins were detected. The results showed that the three *AoErg11s* were expressed differently at different growth times and under different abiotic stresses. All of the three proteins were located in endoplasmic reticulum. The *AoErg11s* could not restore the temperature-sensitive phenotype of *S. cerevisiae erg11* mutant. Overexpression of the three *AoErg11s* affected both growth and sporulation, which may be due to the effect of *AoErg11s* on ergosterol content. Therefore, this study revealed the functions of three *AoErg11s* and their effects on the growth and ergosterol biosynthesis of *A. oryzae*, which may contribute to the further understanding of the ergosterol biosynthesis and regulation mechanism in this important filamentous fungus, *A. oryzae*.

## Introduction

Cytochrome P450 (CYP450), a class of monooxygenases, is a large family of self-oxidizing ferrous heme proteins (Rudolf et al., 2017) and it is named for its unique absorption peak at 450 nm when it binds with CO (Luthra et al., 2011). CYP450 was first discovered in the microsomes of rat liver cells in 1958 (Klingenberg, 1958). In recent years, researchers revealed that CYP450 widely exists in animals, plants and microorganisms (Elfaki et al., 2018), and it is one of the most abundant catalytic enzymes with the widest substrate spectrum in nature. CYP450 participates in the metabolism of endogenous and exogenous substances, including drugs and environmental compounds (Gao et al., 2017; Neunzig and Bernhardt, 2018) and has important effects on cytokines and thermoregulation (Ruparel et al., 2012). CYP450 is mainly distributed in endoplasmic reticulum (ER) and mitochondrial intima (El-Sherbeni and El-Kadi, 2014). As a terminal oxygenase, CYP450 participates in sterol biosynthesis and other processes *in vivo*.

Sterol 14α-demethylase (also named P450_14DM_, CYP51 or ERG11) belongs to the CYP450 superfamily and it is the only P450 enzyme that existed in all organisms including bacteria, fungi, lower eukaryotes, higher plants and mammals (Ghosh, 2017). CYP51 is a very important rate-limiting gene in *S. cerevisiae*, and a key enzyme in the sterol biosynthesis pathway (Jordá and Puig, 2020), catalyzing the substrate 14α-methyl hydroxylation to biosynthesis of sterol precursor (Ogris et al., 2021). Generally, the reaction is reported to occur in the ER; however, it is also reported this reaction might take place in mitochondria in *Kinetoplastidae* (Peña-Diaz et al., 2004). CYP51 is one of the key enzymes in the sterol biosynthesis pathway and final products of the pathway were different in different eukaryotes, such as cholesterol in animals, phytosterols in plants, and ergosterol in fungi. In most situations, sterols are important components of the plasma membrane or precursor of hormone (Zhang et al., 2019). Therefore, lacking this gene causes damage to membrane structure or function, which may eventually lead to the death of fungal, or affect plant growth and development process, or reducing animal the endogenous cholesterol synthesis, etc. Thus, CYP51 became important target for many antifungal drugs, herbicides and cholesterol-lowering drugs (Lepesheva and Waterman, 2011; Zhang et al., 2019; Kaluzhskiy et al., 2021).

CYP51 was first purified from *S. cerevisiae* in 1984 (Yoshida and Aoyama, 1984). As it is an essential enzyme for ergosterol biosynthesis in fungus, the encoding gene was also named *Erg11*. Studies have shown that CYP51 is essential for the growth and development of animals, plants, fungi, etc. In mammals, it is a membrane-bound protein in liver microsomes that plays a crucial role in lipid metabolism, hormone synthesis and detoxification. For example, in mouse, inactivation of the liver CYP51 enzyme leads to the accumulation of lanosterol and 24, 25-dehydrolanosterol, causing progressive liver pathology (Lorbek et al., 2015; Urlep et al., 2017). Besides, CYP51 is also important for mammalian reproduction as sterols are required for activation of oocyte meiosis, and lanosterol 14α-demethylase (FF-MAS) and sterol 14-reductase (T-MAS) were able to reactivate meiosis in mouse oocytes *in vitro* (Byskov et al., 1995). In plants, CYP51 is essential for regulating plant growth and development. For example, *Arabidopsis thaliana* genome contains two *cyp51* genes, *cyp51A1* and *cyp51A2*. The *cyp51A1* is expressed in root tissue, while *cyp51A2* is ubiquitously expressed. Deletion of *cyp51A2* showed various defects, such as hypocotyl dysplasia, short root, reduced cell elongation and seedling mortality (Kim et al., 2005). Besides, *cyp51A2* mutants also show a defective stomatal development phenotype (Qian et al., 2013). In fungi genome, including *S. cerevisiae*, *Candida albicans* or *Cryptococcus neoformans*, it contains only one *CYP51* gene. It has been shown that *CYP51* gene is necessary for aerobic viability of yeast (Bard et al., 1993; Geber et al., 1995; Sanglard et al., 2003; Revankar et al., 2004). Most filamentous fungi contain multiple copies of *CYP51* genes, and these genes usually showed functional redundancy. The deletion of all these *CYP51* genes was fatal for the survival of the cell. For example, *Aspergillus fumigatus* genome contains two *CYP51* genes named *CYP51A* and *CYP51B*. The single deletion mutant of these two genes showed no significant growth defects, while simultaneous deletion of both *CYP51* genes is lethal (Roundtree et al., 2020). Moreover, CYP51A and CYP51B proteins are functionally compensatory. The expression of CYP51A was increased when CYP51B was absent, and *vice versa* (Hu et al., 2007). Similarly, *Magnaporthe oryzae* genome contains two *CYP51* genes, *CYP51A* and *CYP51B*. Single deletion strains of *CYP51A* or *CYP51B* showed no differences in morphology from wild-type strains on CM medium, while simultaneous knockout of both genes is lethal (Yan et al., 2011). It is also revealed that the expression level of CYP51A is significantly increased in *CYP51B* mutant. Unlike the previous two, *Aspergillus flavus* genome contains three *CYP51* genes, *CYP51A*, *CYP51B* and *CYP51C*, which *CYP51A* and *CYP51B* are the major expressed genes for 14α-demethylase activity, since *CYP51C* basal expression is very low or undetectable (Paul et al., 2018). *CYP51A* is the main gene responsible for drug resistance (Lucio et al., 2020), and *CYP51B* is a functionally redundant gene. Some authors have argued that *CYP51C* originated from the duplications of both genes, *CYP51A* or *CYP51B* depending on the species (Pérez-Cantero et al., 2020), and have proposed that *CYP51* duplications are derived from an evolutionary mechanism controlling adaptation to azole toxicity (Hawkins et al., 2014; Dudakova et al., 2017).

However, the function of the *CYP51*/*Erg11* (hereafter named *Erg11*) gene in *A. oryzae*, one of the most important filamentous fungi in industry, has been poorly studied. *A. oryzae* is a filamentous fungus approved by FDA and WHO for safe production. It has long been used not only in traditional food fermentation, brewing and condiment industries but also in modern biotechnology industries such as enzyme preparation and recombinant protein production (Merz et al., 2015; Wang et al., 2021). Unlike reported in yeast, our previous bioinformatics studies have shown that there are three genes encoding Erg11 in *A. oryzae* genome (Hu et al., 2019). However, the function of these three AoErg11s remains unclear. This study investigated the function of the three genes by yeast complementation and examined the expression patterns/subcellular localization of these *AoErg11s* encoding genes/proteins. Finally, the influence of overexpression of these *AoErg11s* on ergosterol synthesis was also examined. This study revealed the function of three *Erg11s* in *A. oryzae* and their effect on growth and ergosterol biosynthesis.

## Materials and methods

### Phylogenetic analysis and functional motifs prediction

Neighbor-joining method was used to create the unrooted tree using MEGA-X, and MEME program was used to identify the conserved motifs of all proteins. The amino acid sequence used is as follows, sterol 14α-demethylase: *A. flavus* [KOC13200.1], *A. flavus* [KOC15064.1], *A. flavus* [KOC13803.1], *A. niger* (XP_001394224.1), *A. niger* (XP_001396151.2), *A. nidulans* (XP_659505.1), *A. nidulans* (XP_681552.1), *S. cerevisiae* (NP_011871.1), *C. albicans* [ADI76627.1], *H. sapiens* [BAG36881.1], *C. imitator* (XP_017392004.1), *M. musculus* (NP_064394.2), *A. thaliana* [OAP10887.1], *A. thaliana* (NP_172633.1), *O. sativa* (XP_015617432.1), *Z. mays* [PWA33212.1]. The accession numbers of *A. oryzae* sterol 14α-demethylase, AoErg11A–AoErg11C, are as follows: EIT83124.1, EIT73378.1 and EIT72345.1. The accession numbers are in parentheses.

### Strains and growth conditions

The wild-type strain (*A. oryzae* 3.042 (CICC 40092)) obtained from China Center of Industry Culture Collection (Beijing, China) and the uridine/uracil auxotrophic (*ΔpyrG*) *A. oryzae* 3.042 strain constructed in our laboratory (Sun et al., 2019a) were used in this study. CD medium (2% glucose, 0.2% NaNO_3_, 0.1% KH_2_PO_4_, 0.05% MgSO_4_, 0.05% NaCl, 0.05% KCl, 0.002% FeSO_4_, 1.5% agar, pH 5.5) supplemented with uracil and uridine was used to collect *A. oryzae* 3.042 *ΔpyrG* conidia suspensions for *Agrobacterium*-mediated transformation. *A. oryzae* was cultured at 30°C for 72 h except where otherwise mentioned. The plasmid constructed with *Escherichia coli* DH5α was transformed into *A. oryzae* using *Agrobacterium tumefaciens* AGL1. Both *E. coli* and *A. tumefaciens* were cultured in Luria Bertani (LB) medium supplemented with appropriate antibiotics at 37°C and 28°C, respectively. All strains used in this study are shown in Table 1, and all plasmids used in this study are shown in Table 2.

**TABLE 1:** List of strains used in this study.

Strain	Brief description	References/Source
*Escherichia coli* DH5α	Cloning of plasmid vectors	Purchased from TransGen Biotech (Beijing, China)
*Agrobacterium tumefaciens* AGL1	Agrobacterium-mediated transformation	Purchased from TransGen Biotech (Beijing, China)
*A. oryzae* 3.042 (CICC 40092)	Wild type *A. oryzae*	Obtained from China Center of Industry Culture Collection (Beijing, China)
Uridine/uracil auxotrophic *A. oryzae* 3.042	*ΔpyrG* auxotrophic *A. oryzae*	Sun et al. (2019a)
*CK*	*ΔpyrG* auxotrophic *A. oryzae* transformed with *pEX2B-DsRed*	Constructed in this study
*AoErg11A* overexpression strain	Transformant of *pEX2B-AoErg11A-DsRed*	Constructed in this study
*AoErg11B* overexpression strain	Transformant of *pEX2B-AoErg11B-DsRed*	Constructed in this study
*AoErg11C* overexpression strain	Transformant of *pEX2B-AoErg11C-DsRed*	Constructed in this study
*AoErg11A* ^*ΔSP*^ overexpression strain	Transformant of *pEX2B-AoErg11A* ^*ΔSP-*^ *DsRed*	Constructed in this study
*AoErg11B* ^*ΔSP*^ overexpression strain	Transformant of *pEX2B-AoErg11B* ^*ΔSP-*^ *DsRed*	Constructed in this study
*AoErg11C* ^*ΔSP*^ overexpression strain	Transformant of *pEX2B-AoErg11C* ^*ΔSP-*^ *DsRed*	Constructed in this study
BY4741(*pYES2.0/*WT)	Wild type, the *S. cerevisiae* control	Purchased from EUROSCARF
Y40975 (*pYES2.0/erg11*)	The *S. cerevisiae erg11* mutant	Purchased from EUROSCARF
*AoErg11A/erg11*	Transformant of *pYES2.0-AoErg11A* to the *S. cerevisiae erg11* mutant	Constructed in this study
*AoErg11B/erg11*	Transformant of *pYES2.0-AoErg11B* to the *S. cerevisiae erg11* mutant	Constructed in this study
*AoErg11C/erg11*	Transformant of *pYES2.0-AoErg11C* to the *S. cerevisiae erg11* mutant	Constructed in this study
*AoErg11A* ^*ΔSP*^ */erg11*	Transformant of *pYES2.0-AoErg11A* ^*ΔSP*^ to the *S. cerevisiae erg11* mutant	Constructed in this study
*AoErg11B* ^*ΔSP*^ */erg11*	Transformant of *pYES2.0-AoErg11B* ^*ΔSP*^ to the *S. cerevisiae erg11* mutant	Constructed in this study
*AoErg11C* ^*ΔSP*^ */erg11*	Transformant of *pYES2.0-AoErg11C* ^*ΔSP*^ to the *S. cerevisiae erg11* mutant	Constructed in this study

**TABLE 2:** List of plasmids used in this study.

Plasmid	Brief description	References/Source
*pYES2.0*	Yeast expressing vector: containing galactose-induced GAL1 promoter	Purchased from Invitrogen
*pYES2.0-AoErg11A*	Yeast expressing vector containing *A. oryzae AoErg11A* gene	Constructed in this study
*pYES2.0-AoErg11B*	Yeast expressing vector containing *A. oryzae AoErg11B* gene	Constructed in this study
*pYES2.0-AoErg11C*	Yeast expressing vector containing *A. oryzae AoErg11C* gene	Constructed in this study
*pYES2.0-AoErg11A* ^*ΔSP*^	Yeast expressing vector containing *A. oryzae AoErg11A* without signal peptide	Constructed in this study
*pYES2.0-AoErg11B* ^*ΔSP*^	Yeast expressing vector containing *A. oryzae AoErg11B* without signal peptide	Constructed in this study
*pYES2.0-AoErg11C* ^*ΔSP*^	Yeast expressing vector containing *A. oryzae AoErg11C* without signal peptide	Constructed in this study
*pEX2B-DsRed*	Binary transformation vector: with *PamyB* as the promoter, *DsRed* was the reporter gene, *pyrG* was the selective marker	Nguyen et al. (2017)
*pEX2B-AoErg11A-DsRed*	Overexpressing *AoErg11A* gene in *A. oryzae*	Constructed in this study
*pEX2B-AoErg11B-DsRed*	Overexpressing *AoErg11B* gene in *A. oryzae*	Constructed in this study
*pEX2B-AoErg11C-DsRed*	Overexpressing *AoErg11C* gene in *A. oryzae*	Constructed in this study
*pEX2B-AoErg11A* ^*ΔSP-*^ *DsRed*	Overexpressing *AoErg11A* ^*ΔSP*^ gene in *A. oryzae*	Constructed in this study
*pEX2B-AoErg11B* ^*ΔSP*^ *-DsRed*	Overexpressing *AoErg11B* ^*ΔSP*^ gene in *A. oryzae*	Constructed in this study
*pEX2B-AoErg11C* ^*ΔSP*^ *-DsRed*	Overexpressing *AoErg11C* ^*ΔSP*^ gene in *A. oryzae*	Constructed in this study
*pEX1-ptrA-GFP*	Binary transformation vector: with *PgpdA* as the promoter, *GFP* was the reporter gene, *ptrA* was the selective marker	Nguyen et al. (2017)
*pEX1-ptrA-ClxA-GFP*	GFP marked endoplasmic reticulum	constructed in this study
*pEX1-ptrA-MTS-GFP*	GFP marked mitochondrial	constructed in this study

### Gene expression analysis

The mycelia at different growth times or under different stress conditions were frozen in liquid nitrogen and crushed immediately after harvest. Total RNA was extracted using a fungal RNA kit (Omega Bio-tek, Norcross, GA, United States) and the cDNAs were synthesized using the Prime Script™ RT reagent kit (Perfect Real Time; Takara). The quality and concentration were determined using a NanoDrop ND-2000 spectrophotometer (Thermo Scientific, Wilmington, DE, United States). All qRT–PCR (quantitative reverse transcription-PCR) operations were performed using SYBR Premix Ex Taq (Takara, Japan) and CFX96 real-time PCR detection system (Bio-Rad, CA, United States). All experiments were repeated three times and the average was taken to calculate gene expression. The housekeeping gene encoding histone H4 as a normalization control (Maruyama et al., 2002), and the relative expression was calculated according to formula 2^−ΔΔCT^. The sequences of the primers used for qRT–PCR are shown in Table 3.

**TABLE 3:** Primers used for qRT–PCR.

Name	Forward (5’→3′)	Reverse (5’→3′)
*rH*	GAC​AAC​ATC​CAG​GGT​ATC​ACT​AAG​C	GGT​CTC​CTC​GTA​GAT​CAT​GGC​A
*qRT-Erg11A*	ACAGCGGCTCTAGTAAGG	TTGAGTTGCCCAAAGGC
*qRT-Erg11B*	CCA​AAG​GAA​CAT​CCA​GTC​C	GTCGCCACAATCACTCC
*qRT-Erg11C*	GTA​CGG​CGA​CAT​CTT​TAC​C	TTCAGCTTGCCATTGAGG

### Functional complementation in yeast

The *erg11* mutant (Y40597) was purchased from EUROSCARF (http://www.euroscarf.de/index.php) and the BY4741 was used as wild-type control. The pYES2.0 vector (Rojas et al., 2011) with *PGAL1* as promoter was used for yeast complementation. Using a one-step cloning kit (Vazyme Biotech Co., Ltd., China), the full-length coding sequence (CDS) of *AoErg11s* and *AoErg11* ^*ΔSP*^ *s* were fused into pYES2.0 vector digested with *HindIII* and *EcoRI*. Then, the constructed vectors were transformed into corresponding yeast mutants using yeast transformation kit II (Coolaber, Beijing, China). The *erg11* mutants were randomly selected and identified by PCR using *S. cerevisiae* and *A. oryzae* ScErg11/AoErg11s-specific primer pairs (primer sequences are listed in Table 4). In temperature-sensitive tests, the control and transformants were grown on YPD (1% yeast extract, 2% peptone, 2% glucose, 1% AGAR) and YPG (1% yeast extract, 2% peptone, 2% galactose, 1% AGAR), respectively, and the phenotypes were evaluated at 30°C and 37°C. In addition, the control and transformants cultured in liquid YPD and liquid YPG for 2 days at 30°C were collected to determine the content of ergosterol.

**TABLE 4:** Primers used for vector construction.

Name	Forward (5’→3′)	Reverse (5’→3′)
*pEX2B-AoErg11A-DsRed-AflII*	TTT​CAC​GTG​CCC​GTG​CTT​AAG​ATG​GGC​ATC​CTA​GCT​GTC​ATT​C	GGA​GGC​CAT​GAT​ATC​CTT​AAG​CGC​CTT​GGT​GAC​AGG​CTC​G
*pEX2B-AoErg11B-DsRed-AflII*	TTT​CAC​GTG​CCC​GTG​CTT​AAG​ATG​ATC​TTC​TCA​CGC​AGC​ATG​G	GGA​GGC​CAT​GAT​ATC​CTT​AAG​TGA​CTT​TTC​TGG​GAA​GCG​TCG
*pEX2B-AoErg11C-DsRed-AflII*	TTT​CAC​GTG​CCC​GTG​CTT​AAG​ATG​TCC​TGG​CCT​CGG​ATT​G	GGA​GGC​CAT​GAT​ATC​CTT​AAG​TCC​CGA​TTT​TGC​AGC​CCG
*pEX2B-AoErg11A* ^*ΔSP*^ *-DsRed-AflII*	TTC​ACG​TGC​CCG​TGC​TTA​AGA​TGA​TTC​TCG​TTG​TGT​CTG​T	GAG​GCC​ATG​ATA​TCC​TTA​AGG​CGA​GCC​TGT​CAC​CAA​GGC​G
*pEX2B-AoErg11B* ^*ΔSP*^ *-DsRed-AflII*	TTC​ACG​TGC​CCG​TGC​TTA​AGA​TGC​GCC​AGC​TCC​TCT​TCC​G	GAG​GCC​ATG​ATA​TCC​TTA​AGT​GAC​TTT​TCT​GGG​AAG​CGT​C
*pEX2B-AoErg11C* ^*ΔSP*^ *-DsRed-AflII*	TTC​ACG​TGC​CCG​TGC​TTA​AGA​TGC​TGA​ACA​AGA​CTA​GGC​C	GAG​GCC​ATG​ATA​TCC​TTA​AGT​CCC​GAT​TTT​GCA​GCC​CGA​C
*pEX1-ptrA-AoclxA-GFP-XhoI*	GCA​GAC​ATC​ACC​CTC​GAG​ATG​CGT​TTC​AAC​GCA​GCT​GTT​G	GGT​ACC​TAC​GTA​CTC​GAG​CTG​GGC​AGA​AGA​ACG​GGT​GGT​A
*pEX1-ptrA-MTS-GFP-XhoI*	GAG​CAG​ACA​TCA​CCC​TCG​AGA​TGG​CTT​CTT​CCT​TGA​GAA​TCG	CTC​ACC​ATG​GTA​CCT​ACG​TAC​TGG​TCG​AGG​GTG​ACC​TCG​C
*pYes2.0-AoErg11A-HindIII*	CTA​TAG​GGA​ATA​TTA​AGC​TTA​TGG​GCA​TCC​TAG​CTG​TCA​T	GAT​GGA​TAT​CTG​CAG​AAT​TCC​GCC​TTG​GTG​ACA​GGC​TCG​C
*pYes2.0-AoErg11B-HindIII*	CTA​TAG​GGA​ATA​TTA​AGC​TTA​TGA​TCT​TCT​CAC​GCA​GCA​T	GAT​GGA​TAT​CTG​CAG​AAT​TCT​GAC​TTT​TCT​GGG​AAG​CGT​C
*pYes2.0-AoErg11C-HindIII*	CTA​TAG​GGA​ATA​TTA​AGC​TTA​TGT​CCT​GGC​CTC​GGA​TTG​G	GAT​GGA​TAT​CTG​CAG​AAT​TCT​CCC​GAT​TTT​GCA​GCC​CGA​C
*pYes2.0-AoErg11A* ^*ΔSP*^ *-HindIII*	CTA​TAG​GGA​ATA​TTA​AGC​TTA​TGA​TTC​TCG​TTG​TGT​CTG​T	GAT​GGA​TAT​CTG​CAG​AAT​TCC​GCC​TTG​GTG​ACA​GGC​TCG​C
*pYes2.0-AoErg11B* ^*ΔSP*^ *-HindIII*	CTA​TAG​GGA​ATA​TTA​AGC​TTA​TGC​GCC​AGC​TCC​TCT​TCC​G	GAT​GGA​TAT​CTG​CAG​AAT​TCT​GAC​TTT​TCT​GGG​AAG​CGT​C
*pYes2.0-AoErg11C* ^*ΔSP*^ *-HindIII*	CTA​TAG​GGA​ATA​TTA​AGC​TTA​TGC​TGA​ACA​AGA​CTA​GGC​C	GAT​GGA​TAT​CTG​CAG​AAT​TCT​CCC​GAT​TTT​GCA​GCC​CGA​C
*ScErg11*	GTC​ATA​TCA​AAC​GTA​CTG​GC	TAC​GTA​CTC​GCA​TGT​ATT​CG

### Gene overexpression

All the gene overexpression experiments were performed using pEX2B (Nguyen et al., 2017), a binary vector with *PamyB* as the promoter. To construct the *pEX2B-AoErg11s-DsRed* and *pEX2B-AoErg11* ^*ΔSP*^ *s-DsRed* vectors, the CDS of *AoErg11s* and *AoErg11* ^*ΔSP*^ *s* were cloned into pEX2B linearized with *AflII*. There was only one enzyme restriction site *EcoRV* between the target gene and *DsRed*, encoding aspartic acid and isoleucine. The primers used in this study are listed in Table 4. All constructed vectors were transformed into *A. tumefaciens* AGL1. Then, the vectors were transformed into *A. oryzae* 3.042 *ΔpyrG* as previously reported (Sun et al., 2019a). At least three individual strains of each transformant were collected to determine the stability of the phenotype, and one of them was selected for the statistical data of three growth experiments. CD medium (with maltose), PDA medium (2% maltose, 20% potatoes, 1.5% agar) and DPY (2% maltose, 1% peptone, 0.5% Yeast Extract, 0.5% KH_2_PO_4_, 0.05% MgSO_4_, 1.5% agar, pH 5.5) was used to culture transformants for phenotypic analysis. Mycelia cultured with DPY medium were collected to determination of ergosterol content.

### Subcellular localization analysis

The AoErg11A-AoErg11C protein localization was predicted by iPSORT prediction website. The constructed *pEX2B-AoErg11s-DsRed* plasmid were transformed into *A. oryzae* 3.042 *ΔpyrG* for subcellular localization. The pEX2B vector with *DsRed* as the reporter gene was used as the control. ER localization protein AoClxA (Watanabe et al., 2007) and mitochondrial localization signal (MTS) (Mabashi et al., 2006) were used as ER localization markers and mitochondrial localization markers. *pEX1-ptrA-GFP* (Nguyen et al., 2017), with *PgpdA* as the promoter and *GFP* as the reporter gene, was used as the co-transformation vector. Then AoClxA and MTS were fused into the vector linearized with *XhoI*, to construct *pEX1-ptrA-ClxA-GFP* and *pEX1-ptrA-MTS-GFP* vectors. There was only one enzyme restriction site *SnaBI* between the target gene and *GFP*, encoding glycine and threonine. To study co-localization, *GFP* vector was transformed into *pEX2B-AoErg11s-DsRed* transformation strain. After the co-transformed strains were obtained, at least three strains were selected for observation, and the most obvious one was selected and cultured on CD medium (with maltose) for 72 h, and the mycelia were observed under 100 x oil microscope with Leica DM4000B microscope (GFP and DsRed fluorescent filter cube, 100 magnification). Primer sequences used to construct plastids are shown in Table 4.

### Measurement of ergosterol

The ergosterol extraction and determination were performed according to previously described methods (Huang et al., 2022). *A. oryzae* mycelia for 72 h were collected and freeze-dried in vacuum to constant weight, and then the mycelia were crushed into powder. Ergosterol was extracted with 50 mg dry powder, and 3 mL ethanolic potassium hydroxide (25 g KOH +35 mL ddH2O, 100% ethanol constant volume to 100 mL) was added to *A. oryzae* powder and vortexed for 1 min. Then incubated at 85°C in a water bath for 1.5 h. After cooling to room temperature, 3 mL of n-heptane (Sigma-Aldrich, St. Louis, MO, United States) and 1 mL of distilled water were added and vortexed for 3 min. The upper layer (n-heptane layer) was separated and stored at 20°C for 24 h before analysis by high performance liquid chromatography (HPLC). HPLC was performed with a Waters Alliance E2695-2489 UV/Vis detector HPLC (Milford, MA, United States) with the UV detector set at 282 nm on a Zorbax SB-C18 column. Methanol/water (95:5, V/V) was used as the mobile phase, and the elution rate was 1.5 mL/min. Ergosterol (Sigma-Aldrich) was used to calibrate the curves. Each experiment was repeated three times.

## Results

### Erg11 is evolutionarily conserved in different organisms

**FIGURE 1:**
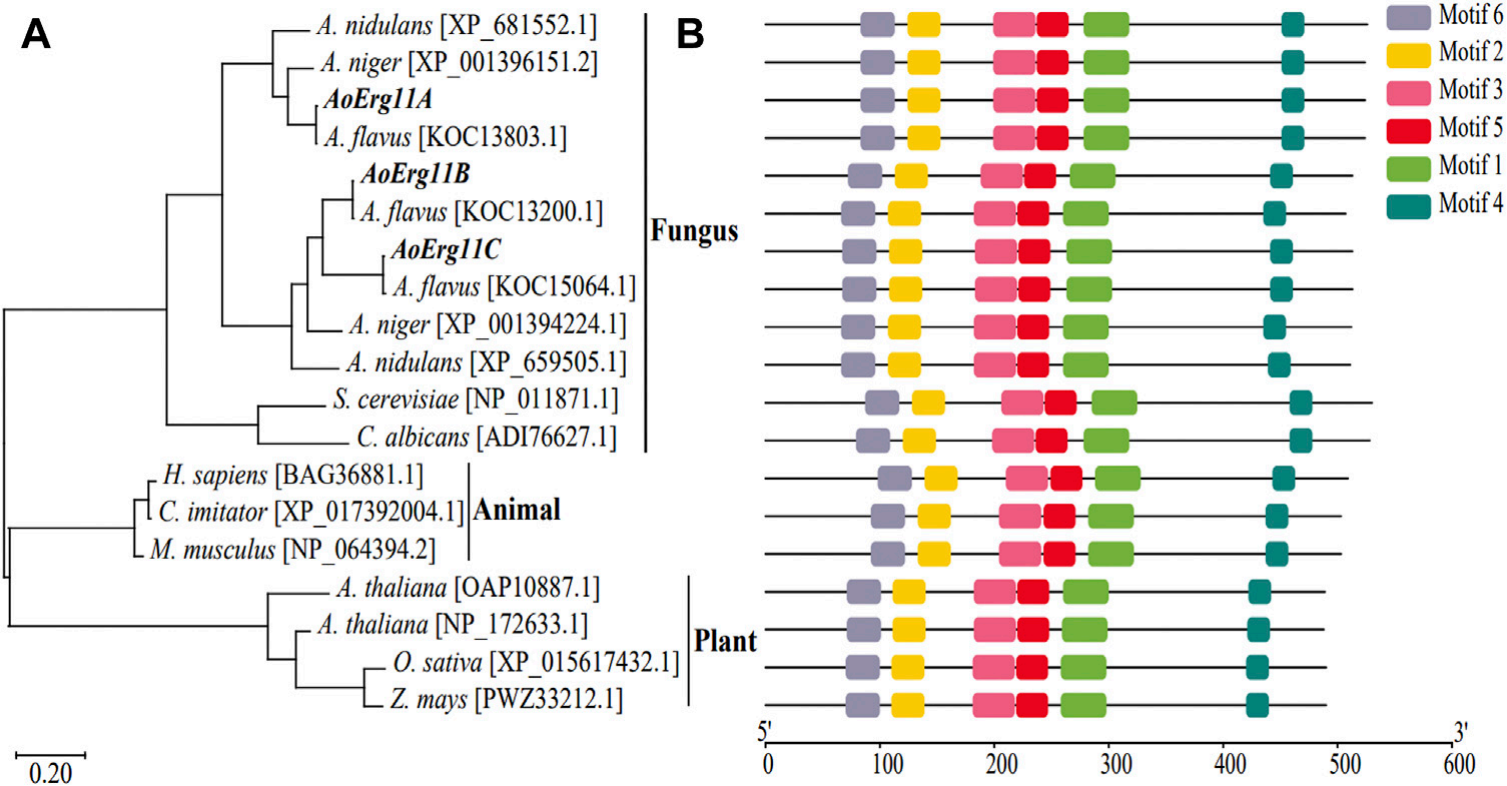
Phylogenetic analysis and functional motifs prediction of Erg11s in different species. **(A)** Unrooted phylogenetic tree of Erg11s and homologous proteins in *A. nidulans*, *A. niger*, *A. flavus*, *S. cerevisiae*, *C. albicans*, *H. sapiens*, *C. imitator*, *M. musculus*, *A. thaliana*, *O. sativa* and *Z. mays*. The IDs of the sequences were included after the specie names in the figure. **(B)** All conserved motifs of the Erg11s were identified by the MEME program. Protein sequences are indicated by thin black line, and the conserved motifs are represented by different colored boxes.

BLAST analysis of corresponding homologous proteins in *A. oryzae* were carried out in NCBI (http://www.ncbi.nlm.nih.gov/) by using *S. cerevisiae* Erg11p sequence as query condition and three homologous proteins (named AoErg11A–AoErg11C) were identified. The AoErg11A protein length is 524 amino acids, while AoErg11B and AoErg11C are both 513 amino acids. To obtain more information about these three AoErg11s, phylogenetic analyses of Erg11 in different organisms were conducted. As shown in Figure 1, the evolution of Erg11 in fungus, animals and plants is relative conserved. In fungus *S. cerevisiae* and *C. albicans* genome harbor only one Erg11, *A. nidulans* and *A. niger* harbor two Erg11, and *A. flavus* and *A. oryzae* harbor three Erg11 (Figure 1A). Motif analysis also showed that all of these Erg11s contain six conserved motifs (Figure 1B). Thus, the Erg11 is evolutionarily conserved across plants, animals and fungi.

### Expression pattern of *AoErg11s*

**FIGURE 2:**
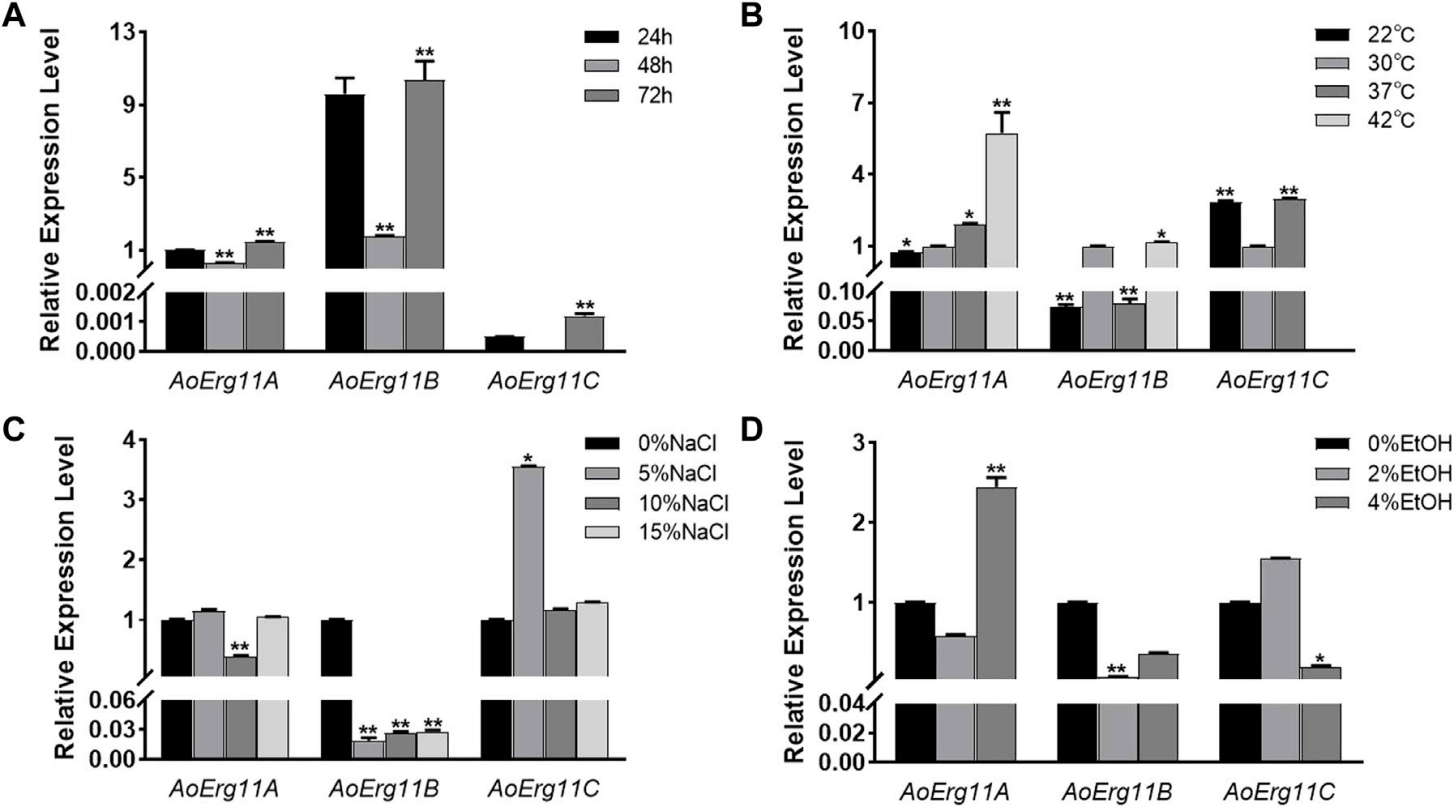
Expression levels of *AoErg11A*–*AoErg11C* at different growth times and under different abiotic stresses on CD medium. **(A)** Expression of *AoErg11A*–*AoErg11C* at 24, 48, and 72 h of growth; **(B–D)** Expression of *AoErg11A*–*AoErg11C* under temperature, salt and ethanol (EtOH) stress conditions. Wild-type *A. oryzae* spores were cultured in CD agar medium alone or CD agar medium supplemented with NaCl or ethanol at 30°C (except for temperature stress). The mycelia were harvested at 24, 48 and 72 h to determine the mRNA levels of *AoErg11A*–*AoErg11C* at different growth times. For other stress experiments, mycelia were collected at 72 h. The expression levels of corresponding genes at 24 h, 30°C and 0% NaCl/ethanol was used as control. Values represent the mean ± SD of three independent experiments. Statistical analyses were performed by *t*-test of GraphPad (*, *p* < 0.05; **, *p* < 0.01). For each experimental group, the relative expression level was compared to the corresponding control.

To investigate the role of *AoErg11*s in *A. oryzae* growth, qRT–PCR was used to determine the expression pattern of *AoErg11s* at different growth times or under different growth conditions. As shown in Figure 2A, under normal conditions, the expression levels of *AoErg11s* differed at different growth times, and *AoErg11B* was the main expressed gene. The expression of *AoErg11A* at 48 h was 1/3 of that in 24 h; and at 72 h, it was 1.5 times of that in 24 h. The expression of *AoErg11B* showed high expression levels at 24 h and 72 h (about 10 times to that of *AoErg11A*) and relative low level at 48 h (about 1/5 of 24 h or 72 h). The expression of *AoErg11C* were very low at 24 h and 72 h: about 1/2000 and 1/1200 to the corresponding *AoErg11A*, while it almost showed no expression at 48 h.

Ergosterol has been reported to participate in the stress response in *S. cerevisiae* (Kodedová and Sychrová, 2015), therefore we also investigated the expression of *AoErg11s* under different stress conditions. *A. oryzae* was stressed by temperature, salt and ethanol. As shown in Figure 2B, under temperature stress, the expression levels of *AoErg11A* gradually increased as temperature increased from 22°C to 42°C. The expression levels of *AoErg11B* were almost the same at 30°C and 42°C, but decreased significantly at 22°C and 37°C, which was about 1/10 of that at 30°C. The expression of *AoErg11C* was tripled at 22°C and 37°C compared with 30°C, and it almost showed no expression under 42°C. Under salt treatment, the expression levels of *AoErg11A* were relative stable, but its expression level reduced to 40% of the control under 10% NaCl (Figure 2C). On the contrary, the expression levels of *AoErg11B* were significantly decreased (about 1/52, 1/37 and 1/35 of the control respectively) by salt treatment (Figure 2C). The expression levels of *AoErg11C* were about the same under 0%, 10% and 15% NaCl, while its expression level increased to about 4 times of the control under 5% NaCl (Figure 2C). Under ethanol stress, the expression of *AoErg11A* decreased by about half under 2% ethanol stress, while increased to over 2 times of the control under 4% ethanol stress. The expression of *AoErg11B* decreased to 8% and 40% of the control under 2% and 4% ethanol stress (Figure 2D). On the contrary, the expression of *AoErg11C* increased 50% under 2% ethanol stress and decreased 80% under 4% ethanol stress compared with the control (Figure 2D). In conclusion, *A. oryzae* possesses three *Erg11* genes, and the expression of these three genes are differed at different growth times and under different abiotic stresses.

### Subcellular localization

**FIGURE 3:**
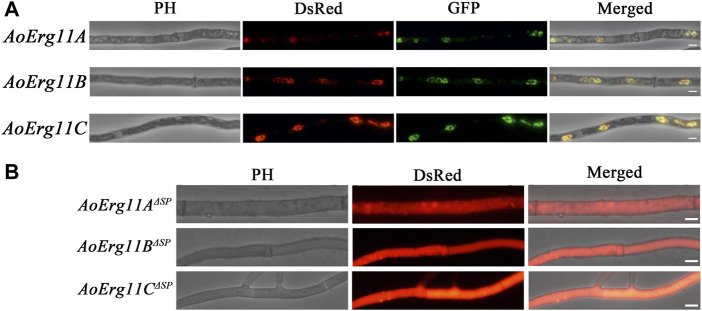
Subcellular localization of AoErg11s. **(A)** Co-localization of *AoErg11s-DsRed* with endoplasmic reticulum. The mycelia of *A. oryzae* 3.042 *ΔpyrG* were co-transformed with *AoErg11s-DsRed* and *AoClxA-GFP* vectors. Left to right: phase contrast, fluorescent image of DsRed, GFP, and merged image of DsRed, GFP and phase contrast. **(B)** The mycelium of *A. oryzae* 3.042 *ΔpyrG* transformed with *AoErg11* ^*ΔSP*^ *s-DsRed*. Left to right: phase contrast, fluorescent image of DsRed, and merged image of DsRed and phase contrast. The scale in the figure represents 5 um.

Sterol 14α-demethylase in *S. cerevisiae* has been reported to be localized in the ER (Jordá and Puig, 2020). However, studies on the subcellular localization of Erg11 homologues in filamentous fungal cells are limited. Bioinformatics prediction analysis showed that there were plant mitochondrial targeted amino acid sequences (MTS) in the N-terminus of AoErg11A, and signal peptide in the N-terminus of AoErg11B and AoErg11C by using iPSORT Prediction website (https://ipsort.hgc.jp/index.html) (Supplementary Figure S1). Therefore, DsRed or GFP was used as reporter protein to investigate the subcellular localization of AoErg11s in *A. oryzae*. The *DsRed* gene was fused into the C-terminal of *AoErg11s*, and the overexpression vectors were constructed with uridine/uracil auxotrophic as the selective marker. Meanwhile, ER (*AoClxA-GFP*)- and mitochondrial (*MTS-GFP*)-targeted *GFP* vectors were constructed with pyrithiamine as selective markers, as reported previously (Sun et al., 2019b). The *AoErg11s* overexpression strains were co-transformed with ER-targeted *GFP* vector and mitochondria-targeted *GFP* vector, respectively. Results showed that the fluorescence of *AoErg11s* overexpression strains had typical ring structures and were consistent with the ER-located GFP marker but not with mitochondria-located GFP marker (Figure 3A and Supplementary Figure S2). In addition, the signal peptides of *AoErg11A*, *AoErg11B* and *AoErg11C* were deleted to construct *AoErg11A* ^*ΔSP*^ *-DsRed*, *AoErg11B* ^*ΔSP*^ *-DsRed* and *AoErg11C* ^*ΔSP*^ *-DsRed* vectors. Microscopic examination showed that red fluorescence signals of *AoErg11* ^*ΔSP*^ *s* overexpression strains were distributed in the cytoplasm uniformity (Figure 3B). Therefore, we concluded that AoErg11A–AoErg11C located in the ER mediated by their signal peptides, and the localization signal of the N-terminal of the protein is related to the localization of these proteins.

### Functional complementation in yeast

**FIGURE 4:**
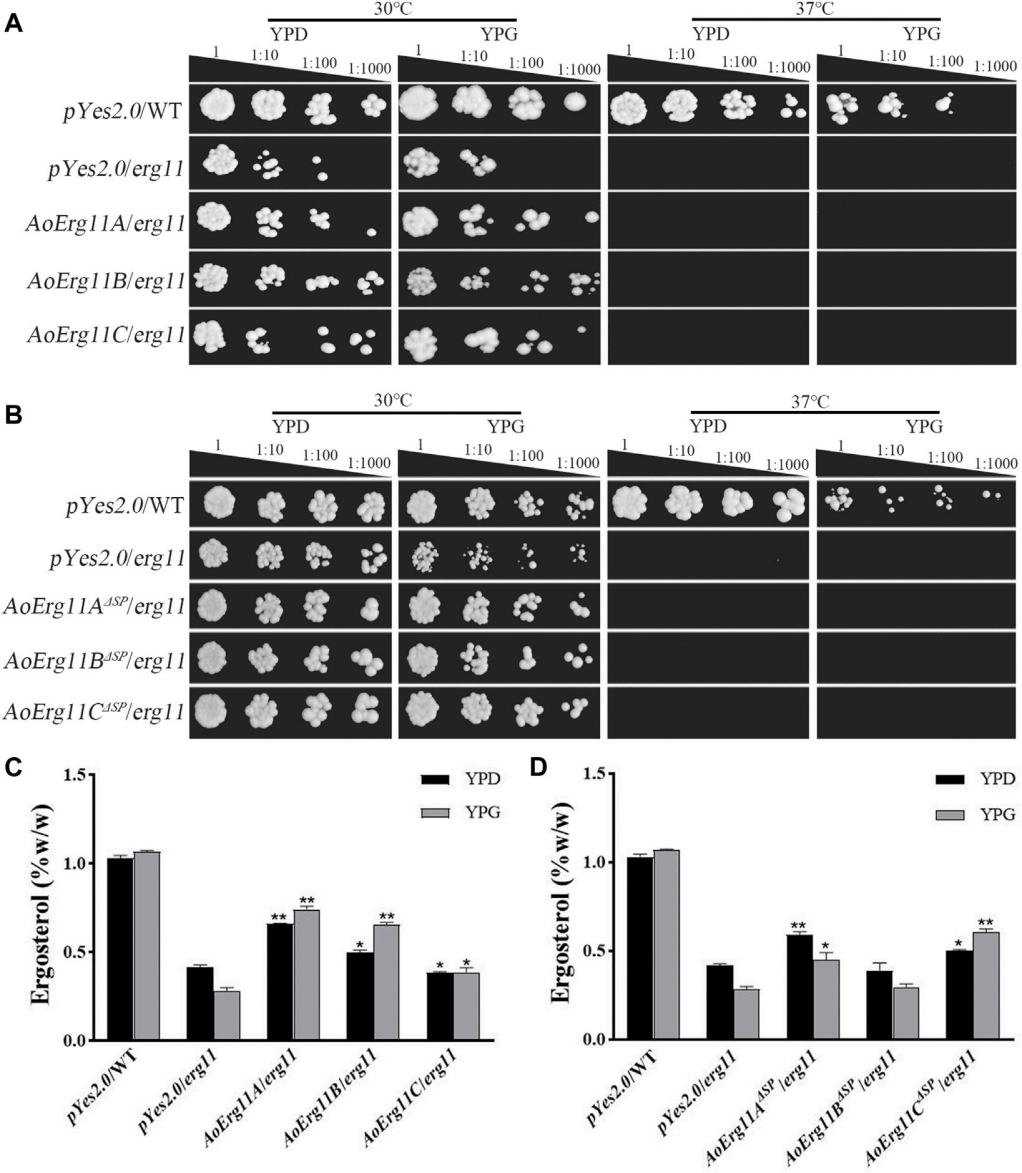
*AoErg11s* and *AoErg11* ^*ΔSP*^ *s* could not recover the phenotypes of the *erg11* mutant of *S. cerevisiae* **(A,B)** Growth of wild type, *erg11* mutant (Y40597), *AoErg11s*/*erg11* transformants and *AoErg11* ^*ΔSPs*^/*erg11* transformants on YPD and YPG medium at 30°C and 37°C. **(C,D)** Measurement of ergosterol contents in all transformants, the control and transformants were cultured in liquid YPD and YPG for 2 days at 30°C, and the yeast were collected to determine the content of ergosterol. Statistical analyses were performed by *t*-test of GraphPad (*, *p* < 0.05; **, *p* < 0.01). The ergosterol content of each experimental group was compared with the *pYes2.0*/*erg11* mutant in the corresponding medium. Values represent the mean ± SD of three independent experiments.

The *S. cerevisiae erg11* mutant was reported to be temperature-sensitive and lethal under 37°C. Thus, *erg11* mutant (Y40597) showing a temperature-sensitive phenotype was used in the yeast heterologous complementary assay. Full length CDS of three *AoErg11*s genes were fused into yeast expression vector (pYES2.0) and transformed into *erg11* mutants. The *ScErg11* gene was also transformed into the *erg11* mutant as a positive control. As pYES2.0 contains a galactose-induced *GAL1* promoter, phenotypes of all the transformants were observed on YPD (with glucose) and YPG (with galactose as an inducer) medium at 30°C and 37°C, respectively. The results showed that *erg11* mutants were lethal at 37°C on YPD and YPG; *AoErg11A/erg11*—*AoErg11C/erg11* could not restore the lethal phenotype of the *erg11* mutants at 37°C while the control can restore the lethal phenotype (Figure 4A and Supplementary Figure S3). Moreover, we deleted the SP sequences of *AoErg11A*–*AoErg11C* to construct *pYES2.0-AoErg11A* ^*ΔSP*^, *pYES2.0-AoErg11B* ^*ΔSP*^ and *pYES2.0-AoErg11C* ^*ΔSP*^ vectors. And transforming them into the *erg11* mutant. Similarly, *AoErg11A* ^*ΔSP*^–*AoErg11C* ^*ΔSP*^ could not restore the temperature-sensitivity phenotype either (Figure 4B).

In addition, the ergosterol contents of the control and all transformants in 30°C liquid medium were measured. When the transformed genes were induced by YPG, the ergosterol content of *AoErg11A/erg11* and *AoErg11B/erg11* transformants were slightly increased compared with that in YPD, while it is still lower than control and the induction expression of *AoErg11C* in *erg11* background showed no effects on the ergosterol content (Figure 4C). On the contrary, the induction of *AoErg11A* ^*ΔSP*^ and *AoErg11B* ^*ΔSP*^ in *erg11* background slightly decreased ergosterol content, but the expression of *AoErg11C* ^*ΔSP*^ increased the content of ergosterol, which was still lower than control (Figure 4D). Thus, it seems that *AoErg11A/erg11*, *AoErg11B/erg11* and *AoErg11C* ^*ΔSP*^ can partly restore the ergosterol content in *erg11* mutant. However, the partial increment of ergosterol content is not enough to restore the temperature-sensitive phenotype of *erg11* mutant.

### Phenotypes of AoErg11s overexpression strains

**FIGURE 5:**
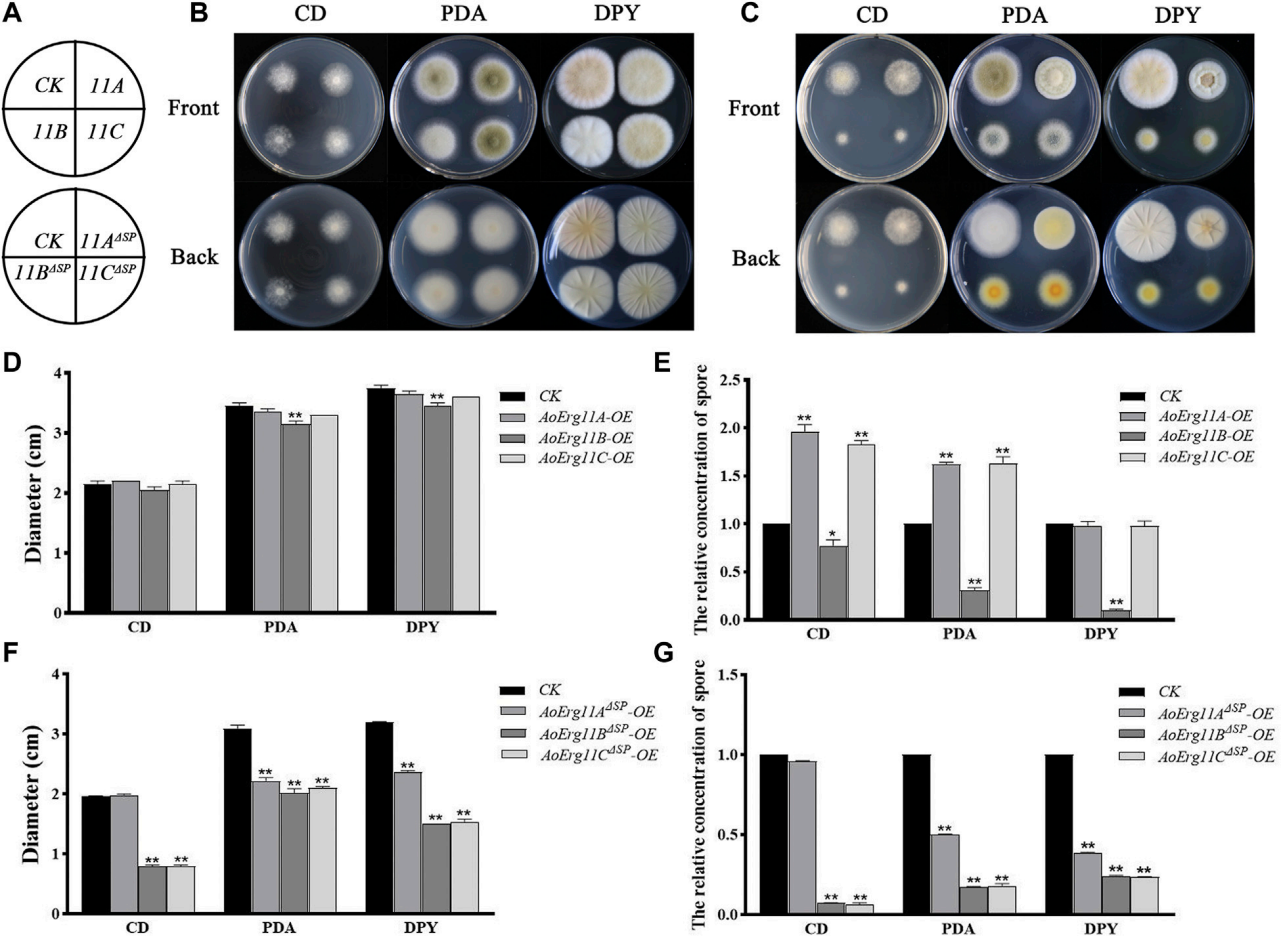
Phenotypes of *AoErg11s* and *AoErg11* ^*ΔSP*^ *s* overexpression strains. **(A)** The schematic of Figure B (upper) and Figure C (lower). **(B,C)** Colony morphologies of CK (wild-type *A. oryzae* transformed pEX2B vector), *AoErg11s* and *AoErg11* ^*ΔSP*^ *s* overexpression strains on CD, PDA and DPY medium after 72 h incubation **(D–G)** The relative concentration of spores and colony diameter of different transgenic strain colonies. Spore suspension of identical concentrations of different *A. oryzae* strains were plated on CD, PDA and DPY agar medium and incubated at 30°C for 72 h. Values represent the mean ± SD of three independent experiments. Statistical analyses were performed by *t*-test of GraphPad (*, *p* < 0.05; **, *p* < 0.01). Each experimental group was compared to the corresponding CK.

The phenotypes of ER- and cytoplasm-located *AoErg11s* overexpressed strains were also investigated. All *AoErg11s* constructs (including *AoErg11A-DsRed*–*AoErg11C-DsRed* and *AoErg11A* ^*ΔSP*^ *-DsRed*–*AoErg11C* ^*ΔSP*^ *-DsRed*) were transformed into *A. oryzae* to obtain the overexpressed strains. Then, these transformants were cultured in CD, PDA and DPY medium (Figures 5A–C). The results showed that there were no significant differences in colony morphology and diameter of *AoErg11A* and *AoErg11C* between overexpressing strains and CK (Figures 5B,D), while the spore numbers in CD and PDA medium increased about 2 times compared with the control (Figure 5E). However, *AoErg11B* overexpressing strains showed significant differences in both colony diameter and spore numbers compared with the control (Figures 5D, E). The colony morphology, diameter and spore numbers of *AoErg11A* ^*ΔSP*^–*AoErg11C* ^*ΔSP*^ overexpression strains were also detected (Figures 5F,G). Compared with the control, the colony morphology, diameter and spore numbers of these overexpression strains were significantly different (except *AoErg11A* ^*ΔSP*^ overexpression strains in CD medium). In conclusion, overexpression of *AoErg11s* could affect the growth and sporulation of *A. oryzae*, and further indicates that the localization of AoErg11s is closely related to its function.

### Ergosterol contents in AoErg11s overexpression strains

**FIGURE 6:**
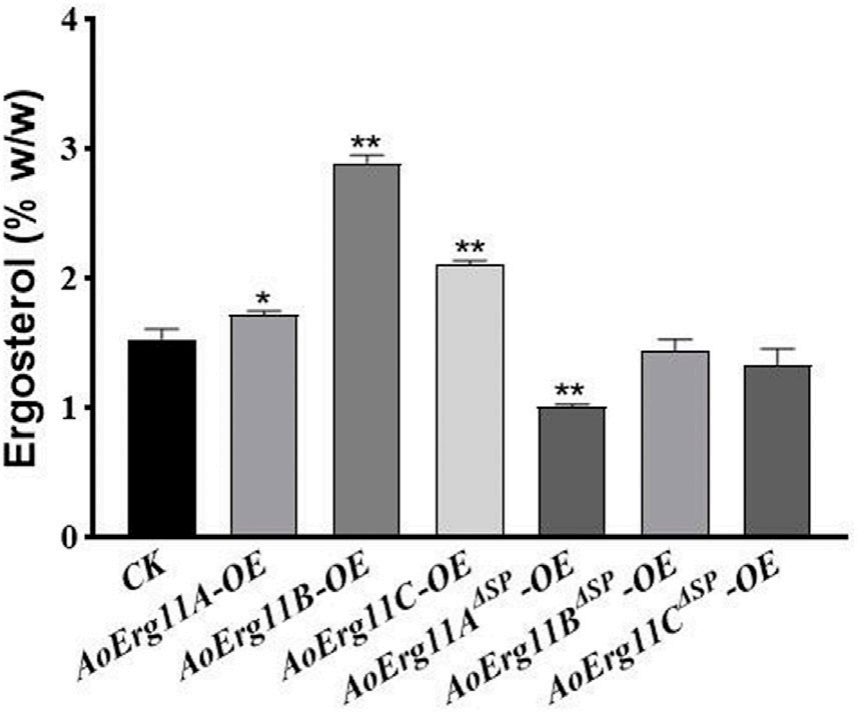
Ergosterol contents of *AoErg11s* and *AoErg11* ^*ΔSP*^ *s* overexpression strains. The control and transformants were cultured in DPY for 3 days at 30°C and collected to determine the content of ergosterol. Values represent the mean ± SD of three independent experiments. Statistical analyses were performed by *t*-test of GraphPad (*, *p* < 0.05; **, *p* < 0.01). The ergosterol content of each experimental group was compared with CK.

Since CYP51 is a key enzyme in the sterol synthesis pathway, therefore we detected the ergosterol content of all *AoErg11s* and *AoErg11* ^*ΔSP*^ *s* transgenic strains. As shown in Figure 6, compared with the control strain, the ergosterol content of *AoErg11s* overexpressing strains increased by 13%, 89% and 38%, while the ergosterol content of *AoErg11* ^*ΔSP*^ *s* overexpressing strains decreased by 34%, 6% and 13%, respectively. Ergosterol content was increased in all the *AoErg11s* overexpressed strains, indicating that *AoErg11s* gene expression was positively correlated with ergosterol synthesis. The ergosterol content of *AoErg11* ^*ΔSP*^ *s* overexpressing strains decreased slightly (except *AoErg11A* ^*ΔSP*^ overexpressing strains), which indicated that AoErg11s localization was closely related to its function. It is consistent with the previous results obtained by subcellular localization. In conclusion, overexpression of AoErg11s disrupts the balance of ergosterol biosynthesis, thus affecting their cellular content.

## Discussion

Previous studies have reported that *Erg11* is an important target enzyme for drug design (Liu et al., 2021). The azole antifungal drugs can bind with the hemoglobin at the active site of Erg11 (Hargrove et al., 2015), thus inhibiting the activity of Erg11, reducing the synthesis efficiency of ergosterol, causing varying degrees of damage to the integrity of the fungal plasma membrane, and suppressing the growth and reproduction of fungi. It has been found that the mutation or high expression of *Erg11* gene is the main reason for the formation of drug resistance in fungi (Sun et al., 2014; Wei et al., 2015). Thus, a large number of studies focus on the discovery of *Erg11* gene resistance mutation sites and the development of novel antifungal drugs with high efficiency and low toxicity. In this study, the function of three AoErg11s from *A. oryzae*, one of the most important industrial fungi, was analyzed. The results showed that: the three *AoErg11s* expressed differently at different growth times and under different abiotic stresses. Under normal conditions, *AoErg11A* and *AoErg11B* is the main expressed gene for sterol 14α-demethylase, *AoErg11C* is almost not expressed. But *AoErg11C* expression was significantly increased under stress conditions, which may indicate that *AoErg11C* plays an important role in *A. oryzae* response to abiotic stresses. All of these three proteins have a signal peptide in the N-terminal, which is localized in ER and distributed uniformly in the cytoplasm after excision of the signal peptide. *AoErg11s* can partially compensate the ergosterol content in *S. cerevisiae erg11* mutant, but cannot completely restore the temperatures-sensitive lethal phenotype. Overexpression of these three *AoErg11s* can affect both growth and sporulation. The number of spores increased significantly after overexpression of *AoErg11A* and *AoErg11C*, while the number of spores decreased significantly after overexpression of *AoErg11B*. Thus, this study revealed the functions of three kinds of Erg11 in *A. oryzae* and their effects on the growth of *A. oryzae* and the biosynthesis of ergosterol, which may contribute to the further understanding of the ergosterol biosynthesis and regulation mechanism in this important filamentous fungus, *A. oryzae*.

### Function of Erg11s in *A. oryzae*

Erg11 was first purified from *S. cerevisiae* (Yoshida and Aoyama, 1984) and has been found successively in animals (Rats) and plants (*Sorghum bicolor*), and is the most widely distributed member of the P450 family (Ghosh, 2017; Elfaki et al., 2018). Motif analysis showed that all of these Erg11s contained six conserved motifs. Therefore, Erg11 is evolutionarily conserved in plants, animals and fungi. There is only one *Erg11* in *S. cerevisiae* genome, two *Erg11s* in *A. niger* and *A. nidulans*, while the third one (*Erg11C*) has evolved in *A. flavus* and *A. oryzae* genome. Previous studies have found that in *A. flavus Erg11A* and *Erg11B* are the main expression genes of 14α-demethylase activity, while the basic expression of *Erg11C* is very low or undetectable (Paul et al., 2018), which is consistent with our results. However, there are also differences between *A. flavus* and *A. oryzae*. *Erg11A* of *A. flavus* is the main gene leading to drug resistance (Lucio et al., 2020), *Erg11B* is a functionally redundant gene, and *Erg11C* is derived from the replication of *Erg11A* (Pérez-Cantero et al., 2020). *Erg11C* does not encode 14α-demethylase, but is essential for the complete virulence of fungus (Fan et al., 2013). In *A. oryzae*, the most important expression gene is *AoErg11B* (Figure 2), and *AoErg11C* is derived from the copy of *AoErg11B* (Figure 1), which plays an important role in *A. oryzae* response to abiotic stresses. We speculate that *AoErg11A* may be a functionally redundant gene. The expression of all three *AoErg11* gene were relative higher at 24 and 72 h, but lower at 48 h. This may be due to the fact that at 24 h, the mycelia of *A. oryzae* is growing and expanding, which requires a large amount of ergosterol to synthesize a complete plasma membrane; at 48 h, the mycelia of *A. oryzae* was relative mature, there requirement of ergosterol was not as necessary as that of 24 h; at 72 h, the proliferation of *A. oryzae* requires a large amount of ergosterol to produce conidia. Our previous studies showed the function of ergosterol biosynthetic pathway genes such as *Erg10* and *Erg19* are conserved between *S. cerevisiae* and *A. oryzae* (Sun et al., 2019a; Sun et al., 2019b). In this study, yeast complementary experiment showed that *AoErg11s* could partially compensate for the ergosterol content in *S. cerevisiae erg11* mutant, but it could not completely restore the temperature-sensitive lethal phenotype of *S. cerevisiae erg11* deletion mutant. In addition, our previous study found that treatment of *S. cerevisiae* and *A. oryzae* with triazolone, an inhibitor targeting *ERG11*, inhibited the growth of *S. cerevisiae*, while it most has no impact on the germination and growth of *A. oryzae* (Hu et al., 2019). All these suggest that the function of the *AoErg11* genes is more complex than that in *S. cerevisiae*. However, because of lacking phenotypes of knockout mutants, the function of these AoErg11s may not be fully uncovered. Further experiments to construct single, double and triple mutants of the three genes should be done to makes their function more explicit.

### Subcellular localization of Erg11s in *A. oryzae*

Previous studies have limited information on subcellular localization of Erg11 in species. In *A. fumigatus*, Erg11A and Erg11B were located in the ER (Roundtree et al., 2020). In *M. oryzae*, Erg11A was mainly located in the cytoplasm of mycelia and conidia (Yan et al., 2011). In this study, we through bioinformatics analysis found that there were plant MTS in the N-terminus of AoErg11A, and signal peptide in the N-terminus of AoErg11B and AoErg11C. Contrary to the predicted results, this study showed that AoErg11A- AoErg11C were located in the ER mediating by the signal peptide. We also noticed that the red fluorescence of *AoErg11A* ^*ΔSP*^–*AoErg11C* ^*ΔSP*^ quenched very quickly (data not shown), indicating that the signal peptides of AoErg11s are not only required for their localization but also important for their homeostasis. Interestingly, the effects on ergosterol contents in ER and cytoplasm located *AoErg11s* over expression *S. cerevisiae* are very different. Phenotypes of overexpressed strains showed that *AoErg11*s could affect mycelia growth or sporulation. Similarly, overexpression of *AoErg11s* and *AoErg11* ^*ΔSP*^ *s* showed different effect on the phenotypes and ergosterol contents. Thus, the function of AoErg11 is closely related to its subcellular localization.

## Data Availability

The datasets presented in this study can be found in online repositories. The names of the repository/repositories and accession number(s) can be found in the article/Supplementary Material.
