# Supplementary material for: The expression pattern, subcellular localization and function of three sterol 14α-demethylases in Aspergillus oryzae
Source: Front Genet. 2023 Jan 23;14:1009746. doi: 10.3389/fgene.2023.1009746 (PMC9899854; doi:10.3389/fgene.2023.1009746)
Supplement: Supplementary file 1 [file Table1.docx]

Supplementary Material

# Supplementary Figures


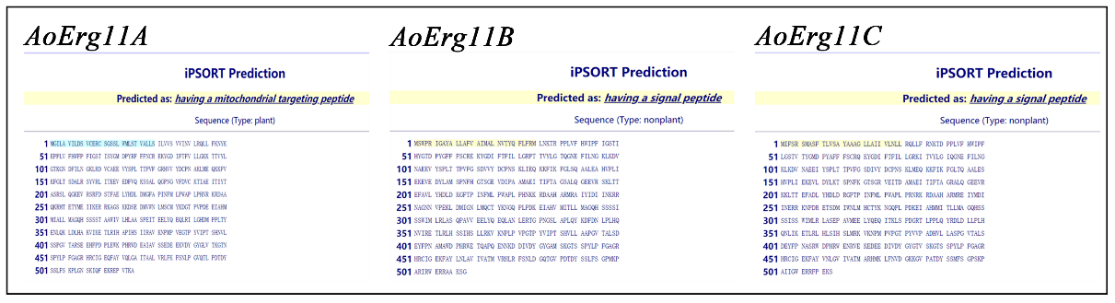


**Supplementary Figure 1.** Subcellular localization prediction of AoErg11s. The protein location prediction website iPSORT Prediction was used. There are plant mitochondrial targeted amino acid sequences (MTS) in the N-terminus of AoErg11A, and signal peptid in the N-terminus of AoErg11B and AoErg11C.

**
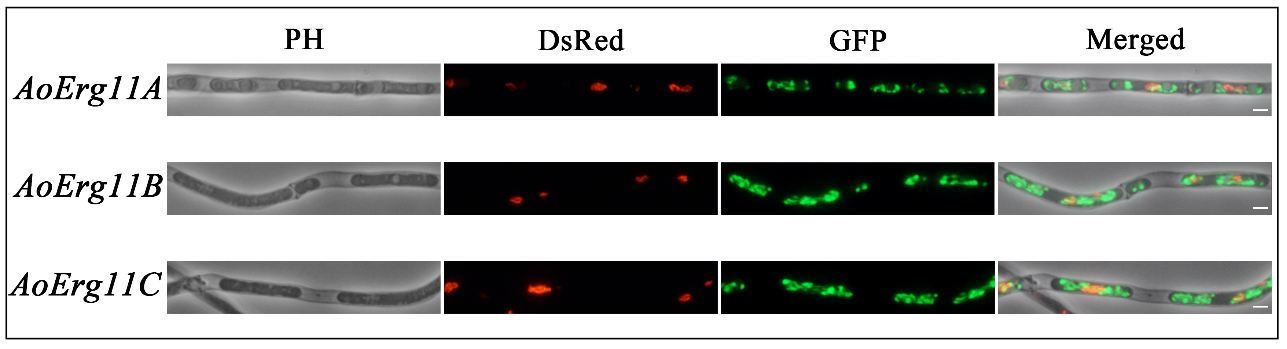
**

**Supplementary Figure 2.** Subcellular localization of AoErg11s. Co-localization of *AoErg11s-DsRed* with mitochondria-localized GFP. The mycelia of *A. oryzae* 3.042 *ΔpyrG* were co-transformed with *AoErg11s-DsRed* and *MTS-GFP* vectors. Left to right: phase contrast, fluorescent image of DsRed, GFP, and merged image of DsRed, GFP and phase contrast. The scale in the figure represents 5 um.

**
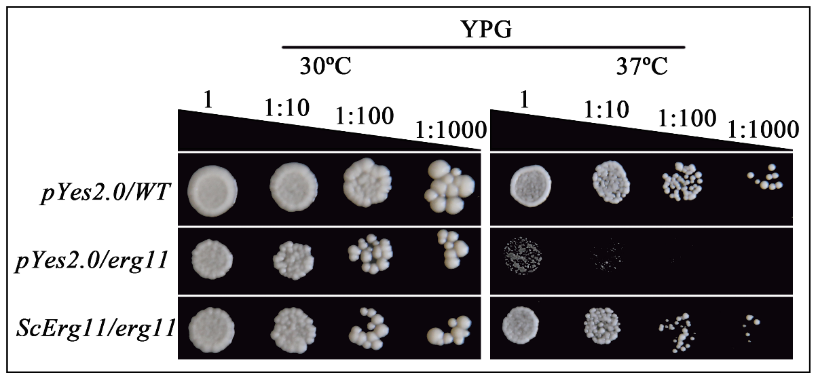
**

**Supplementary Figure 3.** The *erg11* mutant (Y40597) background verification. Wild type, *erg11* mutant, *ScErg11s*/*erg11* transformants were cultured on YPG medium at 30ºC and 37ºC.
